# Deterring non-target birds from toxic bait sites for wild pigs

**DOI:** 10.1038/s41598-021-99547-8

**Published:** 2021-10-07

**Authors:** Nathan P. Snow, Joseph M. Halseth, Justin A. Foster, Michael J. Lavelle, Justin W. Fischer, Michael P. Glow, Ingrid A. Messer, Seth M. Cook, Kurt C. VerCauteren

**Affiliations:** 1 USDA/APHIS/ Wildlife Services, National Wildlife Research Center, 4101 LaPorte Ave., Fort Collins, CO 80521 USA; 2 Texas Parks and Wildlife Department, Kerr Wildlife Management Area, 2625 FM 1340, Hunt, TX 78024 USA; 3 Caesar Kleberg Wildlife Research Institute, Texas A&M University-Kingsville, 700 University Blvd., MSC 218, Kingsville, TX 78363 USA

**Keywords:** Invasive species, Behavioural ecology

## Abstract

Toxic baiting of wild pigs (*Sus scrofa*) is a potential new tool for population control and damage reduction in the US. Field trials testing a prototype toxic bait (HOGGONE 2 containing 5% sodium nitrite [SN]), though, revealed that wild pigs spilled small particles of toxic bait outside of bait stations which subsequently created hazards for non-target species that consumed those particles, primarily passerine birds. To deter non-target birds from consuming particles of spilled bait, we tested four deterrents at mock bait sites (i.e., baited with bird seed) in north-central Colorado, USA during April–May 2020. We found a programable, inflatable deterrent device (scare dancer) reduced bird visitation by an average of 96%. Then, we evaluated the deterrent devices at SN-toxic bait sites in north-central Texas, USA during July 2020, where the devices were activated the morning following deployment of SN-toxic bait. Overall, we found 139 dead wild pigs at 10 bait sites following one night of toxic baiting, which represented an average of 91% reduction in wild pigs visiting bait sites. We found that deterrent devices were 100% effective at deterring birds from toxic bait sites. We found two dead non-target mice at bait sites without deterrent devices. We noted that deploying toxic bait in mid-summer rather than late-winter/early-spring reduced hazards to migrating birds because they were not present in our study area during July. We recommend using deterrent devices (i.e., novel, programmable, battery operated, continuous and erratic movement, and snapping sounds) to reduce hazards to non-target birds at SN-toxic bait sites. We further recommend deploying SN-toxic bait during seasons when migrating birds are not as abundant until further research demonstrates minimal risks to migrating birds.

## Introduction

Wild pigs (*Sus scrofa*; also referred to as feral swine, feral hogs, wild boar, or invasive wild pigs) are one of the most ubiquitous invasive species causing damage to anthropogenic and natural resources worldwide^1–3^. Wild pigs are considered one of the most destructive invasive species to natural environments^4^ and agricultural resources^5,6^. Without more effective tools for controlling wild pig populations, their numbers are predicted to spread and increase in the USA^7,8^, and has potential to increase in other regions of the world^9^. Toxic baits are being developed as a promising new tool to control populations of wild pigs in the USA^10,11^. Additionally, toxic baits are being developed and used in other countries with invasive wild pigs such as Australia^12^ and New Zealand^13^, and are being considered for use in regions with native wild pigs to manage disease risks (e.g., African swine fever)^14^. Despite widespread need, safe and effective application of toxic baits has been challenging because baits must be lethal to wild pigs yet non-lethal for non-target wildlife^15^. Non-target wildlife can be exposed to toxic baits through primary exposure from direct consumption, and secondary exposure from consuming carcasses of wild pigs that were poisoned with toxic bait.

A sodium nitrite (SN)-toxic bait (HOGGONE; Animal Control Technologies Australia Pty) has been designed for wild pigs^10,16^ and was registered for use in Australia in 2019 by the Australian Pesticides and Veterinary Medicines Authority. Risks of secondary hazards to scavengers are low because SN is quickly metabolized after consumption by target species^17–19^. Risks of primary hazards are more concerning because SN is acutely lethal to many species. As such, multiple steps have been taken to reduce non-target exposure to the SN-toxic bait. First, a black colorant was incorporated into the bait matrix to make the bait less attractive to non-target species^16^. Second, a wild pig-specific bait station was developed that relies on ~ 13 kg of magnetic resistance to keep most non-targets from accessing toxic bait^20,21^. The bait station was specifically designed to exclude non-targets while still maximizing access for wild pigs^22^. Despite success of the bait stations, hazards from a SN-toxic bait continued to persist because wild pigs spilled toxic bait outside of the bait stations while feeding^23^. Researchers attempted to mitigate the amount of spilled bait by increasing palatability for wild pigs, compacting bait into bait station compartments to reduce excessive scooping of bait by feeding wild pigs, reducing the concentration of SN from 10 to 5% to minimize residual SN on the landscape, and altering the baiting strategy to minimize training of non-target wildlife to the toxic bait sites^24^. These changes reduced the amount of spilled bait by 19-fold, but still resulted in multiple, small particles of toxic bait (i.e., typically < 5 g) being spilled outside of bait stations, averaging a cumulative ~ 55 g total per bait site^24^. These particles continued to prove hazardous to small non-target species, especially for migratory passerine birds in the grasslands of Texas (TX) during the late-winter/early-spring season^24^.

To further minimize risks to non-target species, we recognized that a deterrent could be useful for abating non-target consumption of SN-toxic bait that is spilled outside of bait stations by wild pigs. A SN-toxic bait is only deployed for ~ 12–24 h at a time and therefore deterrents can remain novel to non-target animals. Also, wild pigs visited bait sites more in the evening or nighttime whereas birds visited in the early morning and daytime^22^, therefore a strategically timed deployment of a deterrent could be targeted to deter birds and not wild pigs. Finally, hazards to birds occurred during a short timeframe; starting pre-dawn when birds became active until an operator arrived at the bait site to remove any spilled bait^24^. Considering these attributes, we hypothesized that a localized and novel deterrent at bait sites could reduce hazards for birds while still allowing undeterred access by wild pigs to toxic bait.

Our first objective was to evaluate four different strategies for deterring birds from bait sites, including two active frightening devices, a physical barrier, and a chemical deterrent. There are numerous active and passive frightening devices available for birds, many of which have had mixed results^25^. However, devices that combined auditory and visual stimuli provided short-term, localized deterrence^26^ which could be sufficient for SN-toxic bait sites. Physical barriers such as netting have been successful at excluding birds from high-value crops^27,28^, and similar barriers could also be used to protect spilled SN-toxic bait. Finally, numerous chemical deterrents containing methyl anthranilate are currently registered in the USA as bird repellents, and have demonstrated repellency on various crops from depredating birds^29–31^. Treating the immediate area surrounding the bait station with such a repellent may deter birds from consuming spilled SN-toxic bait.

Our second objective was to test the physical and chemical deterrents with captive wild pigs to ensure their visitation to bait stations was not negatively impacted. Those deterrents would need to be in place prior to wild pigs accessing toxic bait, which may influence behavior of wild pigs. Our final objective was to evaluate the best deterrent strategy identified above at SN-toxic bait sites with free-ranging wild pigs and birds in north-central TX. We chose this location because this was where hazards to birds were previously reported with the SN-toxic bait^23,24^.

## Study areas

**Figure 1:**
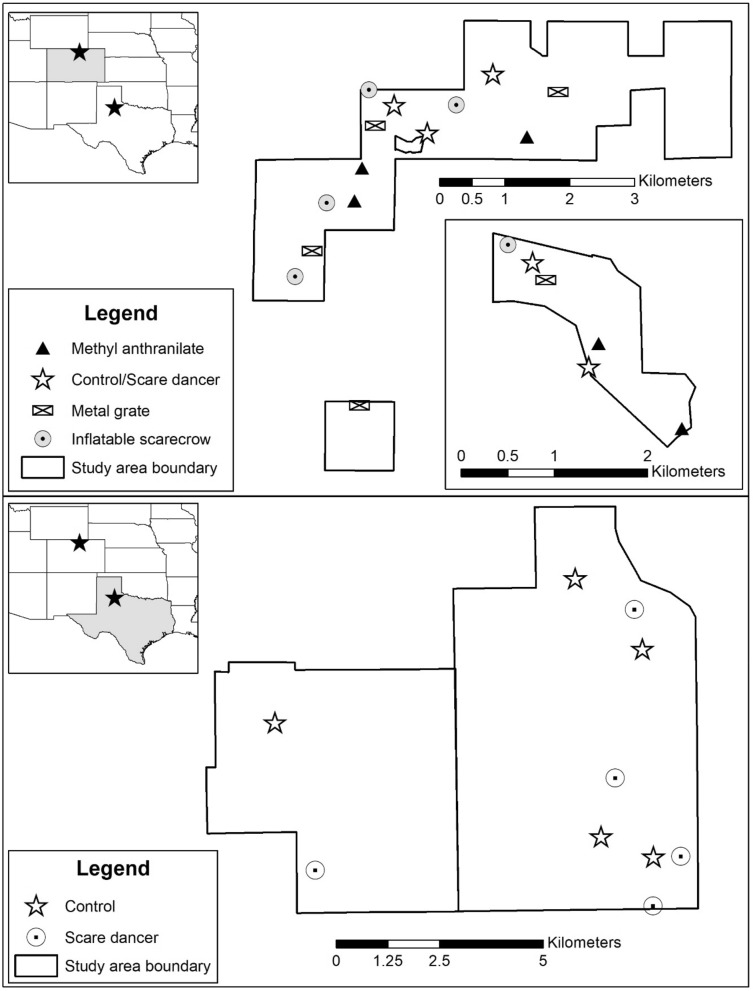
Study areas for evaluating effectiveness of bird deterrents in north-central Colorado, USA during April–May 2020 (top), and for evaluating for reduction in non-target hazards at sodium nitrite toxic bait sites in north-central Texas, USA during July 2020 (bottom).

The first phase of this study was conducted on two sites in north-central Colorado (CO), USA; Wellington State Wildlife Area (SWA) and private property along the Cache la Poudre River near Fort Collins during April–May 2020 (Fig. 1). These study areas are frequented by many species of resident and migrating birds during the spring^32^, including the same species that were encountered in north-central TX in March 2020^24^, which made them ideal for our evaluation. The 7.3-km^2^ Wellington SWA occupied the High Plains ecoregion of CO and was characterized as shortgrass prairie interspersed with constructed wetlands^33^. Dominant vegetation consisted of smooth bromegrass (*Bromus inermis*), tall wheatgrass (*Thinopyrum ponticum*), crested wheatgrass (*Agropyron cristatum*), and cattails (*Typha latifolia*) mixed with hedgerows of juniper (*Juniperus* spp.) and Russian olive (*Elaeagnus angustifolia*). The private property along the Cache la Poudre River was 1.1 km^2^ in the Foothills Shrublands ecoregion^33^. Dominant vegetation consisted of a mix of smooth bromegrass (*Bromus inermis*) pasture and cottonwood (*Populus* spp.)-willow (*Salix* spp.) riparian corridors. Temperatures ranged from − 12.8 to 32.2 °C, and average daily precipitation was 0.04 mm (https://www.wunderground.com/history; accessed 17 Feb 2021).

Captive testing of wild pigs was conducted at the Texas Parks and Wildlife Department, Kerr Wildlife Management Area, in Hunt, TX, USA during April–July 2020. We housed captive-caught wild pigs together in a 2 ha outdoor holding pen for ≥ 2 weeks prior to study initiation. The holding pen was constructed of woven-wire mesh fencing and contained naturally-growing vegetation and trees, and shade structures. Wild pigs were maintained on Bluebonnet 18% Sow Ration Pellet (AC Nutrition, LP, Ardmore, OK, USA) provided at 3–5% of group body mass daily, and water provided ad libitum. Temperatures ranged from 7.2 to 36.1 °C and the average daily precipitation was 0.15 mm (https://www.wunderground.com/history; accessed 17 Feb 2021).

The final phase of this study took place on a 79 km^2^ cattle ranch in north-central TX, USA during July 2020 (Fig. 1). The landscape is categorized as southwestern tablelands^34,35^ comprised of Texas wintergrass (*Stipa leucotricha*) grasslands with mesquite (*Prosopis glandulosa*), juniper (*Juniperus* spp.), and live oak brush (*Quercus virginiana*) (https://tpwd.texas.gov/publications/pwdpubs/media/pwd_mp_e0100_1070n_34.pdf; accessed 17 Feb 2021). The predicted density of wild pigs was estimated at 3–5 pigs/km^2^^8^. Temperatures ranged from 18.3 to 43.9 °C with an average daily precipitation of 0.06 mm (https://www.wunderground.com/history; accessed 17 Feb 2021).

## Methods

### Candidate bird deterrents

**Figure 2:**
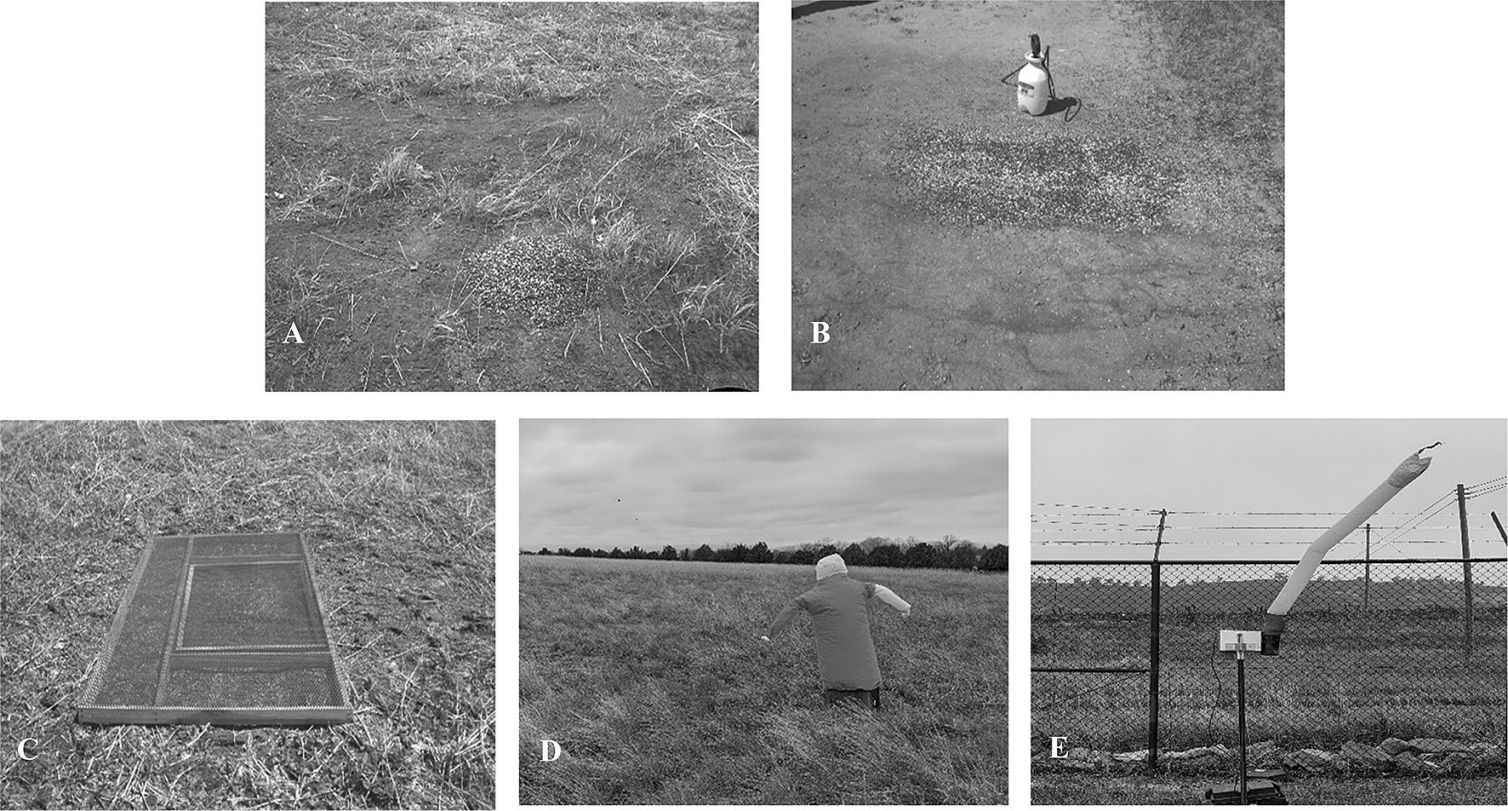
Examples of potential bird deterrents tested in in north-central Colorado, USA during April–May 2020, including (**A**) control, no deterrent, (**B**) 7.5% concentration of methyl anthranilate, (**C**) a metal grate, (**D**), an inflatable scarecrow, and (**E**) a scare dancer. Photos property of USDA.

We identified four candidate bird deterrents that were suitable for deployment within a SN-toxic baiting program (Fig. 2). Specifically, we searched published studies and vendor websites to identify candidate bird deterrents that had a proven record of deterring birds, or features that we expected would deter all birds after a deployment of SN-toxic bait while not deterring wild pigs. These features included: (1) not deterring wild pigs (i.e., user programmable operating hours for after wild pigs visits or being bird-specific), (2) aversive to birds (i.e., erratic movements or irritating to birds), and (3) remotely operated (i.e., battery operated or effects lasting ~ 12 h if user applied).

We selected two frightening devices that offered visual and auditory stimuli, were battery-powered, and programmable to have a user-specified start time. The first frightening device was a 1.8 m inflatable scare dancer (Snake 6 ft Cordless Inflatable Scarecrow, AirCrow LLC, Lake Charles, LA, USA). The scare dancer was a yellow nylon tube shaped like a snake and inflated by a small fan and control unit powered by a 12 V battery connected to a programmable control panel. If using the scare dancer for SN-toxic bait deployment, our strategy would be to program the device to operate continuously starting 1 h before first light the morning after toxic bait was deployed. Our expectation would be that wild pigs would have already visited bait sites and consumed SN-toxic bait prior to scare dancer activation. Once activated, the scare dancer would deter non-targets away from any spilled SN-toxic bait during the morning after toxic baiting until operators arrived to clean the site.

The second frightening device was an inflatable scarecrow called the Scarey Man Birdscarer (Clarratts Ltc, United Kingdom). This device was also powered by a small fan using a 12 V battery, activated by a timer, and inflated for 25 s every 18 min accompanied by an audible 112 db siren. The timing of the inflation could not be altered. The blaze-orange inflatable scarecrow bobbed up and down as it inflated and deflated, and emitted a siren wail. Our strategy with the inflatable scarecrow, following SN-toxic bait deployment, would be the same as the scare dancer, except the inflatable scarecrow could not be programmed to operate continuously.

For the physical barrier treatment, we constructed a metal grate using a 2.4 m × 1.2 m sheet of #13-gauge steel diamond-shaped expanded metal. The maximum openings of the expanded metal were 1.0 cm and were raised (i.e., tapered upwards) to facilitate bait falling through the grate. We constructed the grate to sit 9.0 cm above ground using a frame of standard construction lumber. We also tapered the top of the wooden frame to reduce surface area and facilitate bait falling through the grate. If using the grate for SN-toxic bait deployment, our strategy would be to put the bait station on top of the grate. Our expectation would be that wild pigs would stand on the grate to access the bait station, and spilled particles would fall under the grate and be inaccessible to non-target animals.

The chemical repellent treatment we tested was Avian Migrate™ Goose and Bird Repellent (Avian Enterprises, Jupiter, FL, USA) which contained 14.5% methyl anthranilate. Avian Migrate required dilution with water for all applications. We followed the label instructions for spot repelling, and used the strongest dilution recommended at 50:50 Avian Migrate and water, resulting in 7.5% methyl anthranilate. We used a hand-pump-pressurized garden sprayer to apply 500 ml of the mixture to a 3 × 3 m area which resulted in an even and thorough coating of the area. Aversion to methyl anthranilate may be a learned behavior as an irritant for birds^36^, therefore would need to be applied daily for 1–2 days prior to SN-toxic bating. If using the repellent for SN-toxic bait deployment, our strategy would be to spray the ground immediately surrounding bait stations for 2 nights prior to deploying toxic bait, and the night of toxic baiting. Our expectation would be that by the 3rd night of application non-target birds would be repelled from consuming particles of spilled bait that fell on the treated ground; after which, we could safely deploy SN-toxic bait.

### Field study on deterrent effectiveness for birds

We initially selected and pre-baited ~ 60 sites in north-central CO using 5 kg of bird seed (Deluxe Blend Bird Seed, Wild Birds Unlimited, Fort Collins, CO, USA). Sites were selected in diverse land covers that were likely to hold small passerine birds, such as thickets, wind rows, near water sources, or along shelter belts; and based on distance to nearby sites (i.e., goal of > 500 m to nearest site). We cleared sites of tall grass and debris to ease discovery and access to the bird seed by smaller birds. We visited sites every 2–3 days to replenish and maintain ~ 2 kg of bait at the sites. We pre-baited sites for ~ 4 weeks to ensure birds were well-acclimated to visiting sites daily.

We monitored visitation to sites using remote cameras (RECONYX PC900, RECONYX Inc, Holmen, WI, USA) mounted on T-post approximately 5 m from the bait pile, 1.5 m above ground, and angled down at 70° to provide a consistent field of view at each site. Cameras were programmed to record time-lapse imagery every 2 min (i.e., 720 images/day) which was used to calculate indices of species visitation. We used the Colorado Parks and Wildlife Photo Database to process all time-lapse imagery (Ivan and Newkirk 2016). For each image, a single observer recorded presence and count of each unique species present. We selected the best 20 sites (Fig. 1) based on the greatest rates of bird visitation, greatest diversity of bird species visiting, and lowest presence of other species that consumed large quantities of the bird seed (e.g., raccoons, deer, skunks).

For the trial, we randomly assigned a deterrent treatment (i.e., inflatable scarecrow, metal grate, methyl anthranilate) or control (i.e., no deterrent method) to five sites each. We re-used the control sites to test the scare dancer after testing the initial four treatments, because the scare dancers were received later than first three treatments. We visited bait sites daily and weighed the amount of bird seed remaining to calculate the amount consumed with digital scales (MeasureTek GGS_42964, MeasureTek Scale Co, Ltd, Vancouver, BC, Canada). We replenished each site to ensure ~ 2 kg of fresh bird seed was available each day.

The trials were seven consecutive days (Table 1). We focused on species visitation from 1 h before first light (~ 0500 h) to midday (1200 h) each day, because this time period represented the critical hours in which hazards occurred at toxic bait sites^22,24^. We visited the bait sites between 1200 and 1400 h each day to replenish bait and prepare sites for the following day. The 7-day trial consisted of:

- Days 1–2 = Pre-baiting days. No deterrent deployed.
- Day 3 = Acclimation day. We deployed the deterrent devices but did not activate. Scare dancers were installed on a t-post 1.5 m above the bait sites. Inflatable scarecrows were placed on the ground 3 m away from the bait sites. Metal grates were deployed 3 m away from the bait sites. Methyl anthranilate was sprayed for first time in the 3 × 3 m area surrounding bait sites to initiate the learned repellency.
- Day 4 = Pre-treatment day. This was the day we collected pre-treatment data (i.e., consumption and remote camera data) for comparison with treatment and post-treatment below. All deterrent devices remained inactive as described for acclimation day. The methyl anthranilate was sprayed in the same manner as before for the second time.
- Day 5 = Treatment day. Both frightening devices were activated at 1 h prior to first light. The metal grate was installed over the bird seed. Methyl anthranilate was sprayed in the same manner as before for the third and final time.
- Day 6 = Post-treatment day. All deterrent devices were inactivated but left in place similar to the pre-treatment day. The metal grate was moved 3 m away from the bait site. No methyl anthranilate was sprayed.
- Day 7 = Removal day. We removed all our cameras and deterrent devices and ceased re-baiting at all sites.

**Table 1:** Strategies used to evaluate effectiveness of bird deterrents during a 7-day trial in north-central Colorado, USA during April–May 2020.

Day of study	Baiting stage^a^	Bird-deterrent treatment				
		Control	Scare dancer	Inflatable scarecrow	Metal grate	Methyl anthranilate
1	Pre-bait	Bait	Bait	Bait	Bait	Bait
2	Pre-bait	Bait	Bait	Bait	Bait	Bait
3	Acclimation	Bait	Deployed but inactive	Deployed but inactive	Deployed but inactive	Application 1^b^
4	Pre-treatment	Bait	Deployed but inactive	Deployed but inactive	Deployed but inactive	Application 2^b^
5	Treatment	Bait	Activated^d^	Activated^d^	Activated^c^	Application 3^b^
6	Post-treatment	Bait	Deployed but inactive	Deployed but inactive	Deployed but inactive	Bait
7	Removal	Bait	Deployed but inactive	Deployed but inactive	Deployed but inactive	Bait

^a^For all stages, 2 kg of store-bought bird seed (Wild Birds Unlimited Deluxe Blend, Fort Collins, CO, USA) was deployed daily at each bait site. Any remaining bird seed from the previous day was removed.

^b^A spot treatment application of Avian Migrate (containing 14.5% methyl anthranilate) mixed into 50% water was applied to the ground in a ~ 3 × 3 m area. Then, the 2 kg of bird seed was dispersed over the treated area.

^c^The metal grate was placed over top of the 2 kg pile of bird seed upon deployment until the operators arrived the following day (i.e., ~ 1200 h).

^c^The inflatable scarecrow and scare dancer were programmed to activate starting at 1 h before first light. The devices remained activated until operators arrived to re-deploy bird seed the following day (i.e., ~ 1200 h).

For each site, we calculated an index of the number of passerine birds observed in each two-min time-lapse image (rate = average number of birds/two mins) during morning hours (i.e., 0500–1200) for the morning of pre-treatment, treatment, and post-treatment. We compared indices among each of the 3 days and five treatments using negative binomial mixed models and log-links with package glmmTMB^37^ in Program R v3.6.3^38^. We used offsets of the number of hours monitored and site ID as a random effect to account for repeated (i.e., daily) measures taken at each site. We did not analyze for other species (i.e., predatory birds and mammals) because visitations were rare. For all analyses we calculated and examined the 95% confidence intervals (CIs) surrounding the regression coefficients (β) for non-overlap of zero to indicate statistical and biological differences.

### Effects of deterrents on captive wild pigs

We evaluated whether the deterrents influenced feeding behaviors of captive wild pigs. Specifically, we evaluated how wild pigs responded to the metal grate and methyl anthranilate, because these deterrent strategies would need to be in place as wild pigs visited bait sites, and we wanted to ensure wild pigs would not be deterred from feeding. Contrarily, neither of the deterrent devices should be encountered by wild pigs because these devices would be operated on a timer and set to activate after wild pigs visited toxic baiting sites. Therefore, we did not evaluate those treatments with captive wild pigs.

For testing methyl anthranilate, we randomly selected and placed three captive wild pigs from the larger holding pen (i.e., two males and one female) into three 0.02 ha pens, respectively. We replicated this design twice, for a total of six pens (n = 18 wild pigs) tested. The wild pigs in each pen were acclimated for one night to the new pens and to feeding from two identical feed troughs (1.8 × 0.3 × 0.1 m) placed 3.2 m apart. Each night we fed ~ 10 kg of whole kernel corn in each trough and weighed any remaining corn the following morning. A 2-choice feeding test was conducted on nights two, three, and four, where we applied methyl anthranilate to a 3 × 3 m area surrounding one of the troughs using the same mixture as described above in CO. For the other trough, we did not apply methyl anthranilate to the surrounding soil. We applied the methyl anthranilate and whole kernel corn each evening of the 3-day treatment period.

For testing the metal grate, we randomly selected and placed four captive wild pigs from the larger holding pen into two 0.2 ha pens, respectively. We replicated this design twice, for a total of four pens (n = 16 wild pigs) tested. A single feed trough (1.8 × 0.3 × 0.1 m) was placed in each pen. We placed the metal grate under the trough in one pen where it remained for the three nights of study. Two kg of pelleted sow ration were fed in each pen on night 1. On night two, ~ 10 kg of a placebo SN-toxic bait (i.e., HOGGONE without SN) and 1 kg of pelleted sow ration were fed in each pen. On night three we offered just 10 kg of placebo bait to evaluate whether spilled particles of the peanut paste-based bait^16^ would stick to the metal grate. We ceased testing the metal grate after the second replicate because we observed that wild pigs spilled small particles of the placebo bait which stuck to the top of the metal grate in the first replicate, followed by 100% aversion by wild pigs to the metal grate in the second replicate, rendering the metal grate a non-viable option for operational use.

For the methyl anthranilate, we compared proportions of whole-kernel corn consumed in the 2-choice test using a linear model in Program R. We evaluated the interaction of treatment × night to determine if the application of methyl anthranilate influenced the amount of corn wild pigs consumed over time. We also tested the reduced model without the interaction to best interpret the unconditional main effects^39^. We did not analyze data from the metal grate treatment because the evaluation was stopped early, and the results were clear.

### Field evaluation of deterrent with toxic bait

For the final phase of this study, we evaluated the most effective deterrent identified in the first phase of the study (i.e., scare dancer deterrent device, see results) and implemented this deterrent device into a SN-toxic toxic baiting program for wild pigs in north-central TX. We followed methodologies established in previous studies (Table 2) to initiate a SN-baiting program^24,40–42^. Specifically, we initially deployed ~ 30 bait sites by placing ~ 11 kg of whole-kernel corn on the ground at locations with recent sign of wild pigs (e.g., fresh tracks, feces, wallowing, rooting). We installed one remote camera on a t-post 5 m away from each bait site, 1.5 m above ground, and angled down at 70°. We programmed cameras to capture time-lapse images every 5 min (i.e., 288 images/day). We revisited bait sites every day for 5 days to refresh bait (i.e., maintain 11 kg of corn) and view camera images for wild pigs. After day 5, we selected the 10 best sites (Fig. 1) using the highest ranked sites from this ranking system: (1) consistent wild pig visitation (i.e., ≥ 2 days in a row), (2) consistent visitation by a family group of wild pigs (i.e., ≥ 1 female with multiple juveniles or piglets), (3) consistent visitation by multiple family groups (4) consistent visitation of independent family groups not visiting other sites^42^. We also made sure to select bait sites that were > 500 m apart to maintain independence among the groups of pigs visiting each site^41,43^.

**Table 2:** Baiting strategy to locate, pre-bait, and train wild pigs to use bait stations and consume SN-toxic bait used in north-central Texas, USA during July 2020.

Nights of study^a^	Baiting stage	Bait deployed at each site daily (kg)		
		Whole-kernel corn	Placebo bait	SN-toxic bait
1–5	Pre-baiting on ground—locate wild pigs	10.0	0.0	0.0
6–7^b^	Training—introduce bait stations, lids propped 2.5 cm	10.0	0.0	0.0
8	Training—bait station lids propped 5 cm	7.5	2.5	0.0
9–10	Pre-placebo baiting—bait station lids closed with 13 kg magnetic resistance	0.0	7.5	0.0
11^c^	Toxic baiting—bait station lids closed with 13 kg magnetic resistance	0.0	0.0	7.5
12–13	Post-Placebo baiting—bait station lids closed with 13 kg magnetic resistance	0.0	7.5	0.0

^a^Adjusted ± 2 nights at any stage if wild pigs did not visit during certain nights, needed a longer training period because they did not access, or readily accessed and could advance more quickly. Our goal was to advance through each baiting stage as quickly as possible with the balance of allowing the majority of wild pigs access bait inside of the bait stations at each stage, determined by daily examination of remote camera images.

^b^Bait stations were placed 10–30 m from pre-baiting sites to avoid any particles of whole-kernel corn left on the ground in an effort to reduce non-target visitation.

^c^At 5 of 10 bait sites with the deterrent devices, the devices were activated at 0520 h (i.e., 1 h before first-light) the morning following toxic bait deployment. Deterrent devices were deactivated upon arrival of operators to bait sites and removing any toxic bait spilled outside the bait stations by wild pigs (i.e., ~ 0900–1200 h).

We deployed wild pig-specific bait stations^20^ with ~ 13 kg of magnetic resistance on the lids^21^ at the 10 final sites and initiated a series of conditioning phases to acclimate wild pigs to open and consume bait from inside the bait stations (Table 2). We deployed two bait stations at sites with ≥ 10 wild pigs to ensure all wild pigs had sufficient access to bait. We deployed bait stations 10–30 m away from initial pre-baiting sites (where we originally placed corn on the ground) to reduce visitation by non-target animals that may be attracted to residual particles of corn. Where cattle were present, we also constructed 3-strand barbed-wire fences around the site to exclude them from accessing SN-toxic bait.

**Figure 3:**
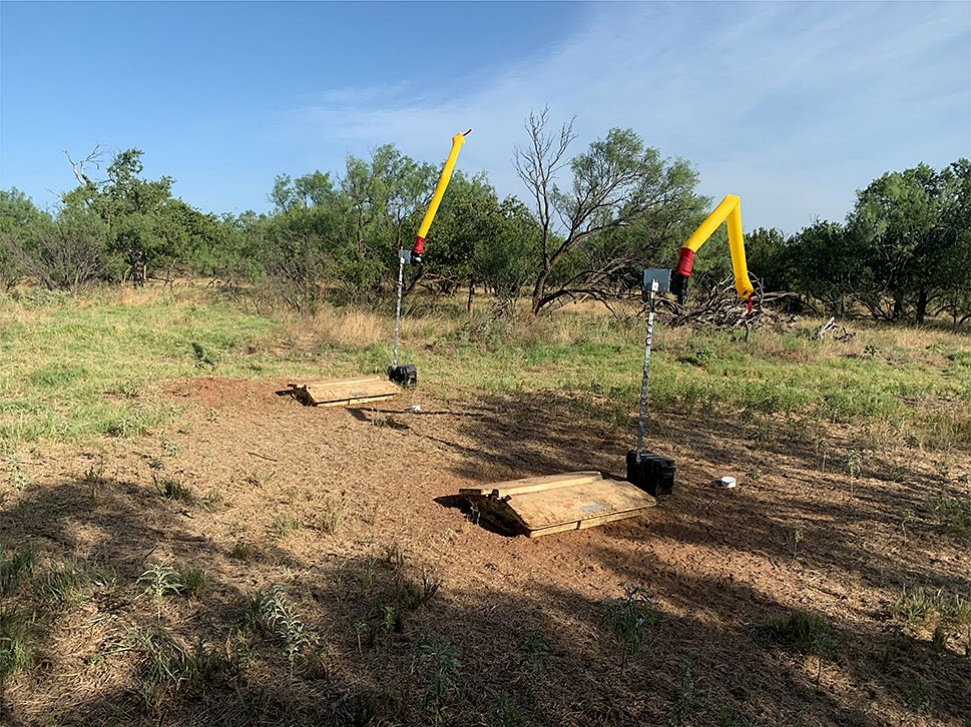
Example of activated deterrent devices (scare dancers) mounted above bait stations containing a sodium nitrite toxic bait in north-central Texas, USA during July 2020. Photo property of USDA.

We randomly selected five sites to deploy the deterrent devices, and five sites as controls (no deterrent devices). Three days prior to deploying SN-toxic bait, we deployed the deterrent devices but left them inactive to condition wild pigs to the presence of the devices. We mounted the deterrent devices on T-posts approximately 1.8 m above ground directly over each bait station with the battery box secured at the base of the T-post (Fig. 3). When we deployed SN-toxic bait, we programmed the deterrent devices to activate at 0520 h the next morning (i.e., 1 h before first-light). We waited until 0900–1200 h the next morning before visiting bait sites to allow ample testing time of the deterrent devices to deter birds, and to simulate realistic use in an operational setting. When we arrived at the bait site, we deactivated the deterrent devices and cleaned the surrounding area of any remaining spilled bait. We collected and weighed all spilled bait we could locate and turned over the soil surrounding the bait station to bury any small particles of spilled bait we could not collect.

We conducted systematic carcass searches along transects following the SN-toxic bait deployment. Specifically, we searched 400 m × 400 m transect grids centered on the bait sites every 50 m, walking transects oriented North/South the first day and East/West the second day. We generated the transects in ArcGIS (v10.8.1, Environmental Systems Research Institute, Redlands, CA, USA), and uploaded them to handheld devices (i.e., mobile phones or tablets) using ArcGIS Explorer (v20.0.1) to navigate along the transects. Additionally, we searched a smaller 50 m × 50 m transect grid centered on the bait sites every 5 m for three consecutive days, again switching between North/South, East/West, and North/South orientation each day, respectively. Transect spacing and distances were based on locations of carcasses found in a previous study with SN-toxic bait^24^. We searched transects for multiple days to ensure any carcasses were located and to determine if any animals succumbed to consuming spilled SN-toxic bait that may have been missed during our clean-up process days after deployment.

We recorded sex, age based on tooth eruption^44^, weight, location, and evidence of SN-toxic bait consumption of any dead wild pigs that we located. Bait consumption was determined by observing bait in the mouth or stomach, or based on the percentage of methemoglobin in the blood by comparing the red-color-value of a drop of blood on a white laminated card to a standard curve^45^. For any non-target animals found dead, we recorded species, location, and evidence of SN-toxic bait consumption (as described above).

We processed all time-lapse imagery from each bait station using the Colorado Parks and Wildlife Photo Database^46^. For each image, a single observer recorded the count of each species present. We did not include cattle because they were excluded from bait sites. We used two indices from the images for comparing the rates of visitation by different species. First, we used an index of the count of non-target animals/image during the hours that the deterrent devices were operating (0520–1200 h). We compared this index among the days of pre-, during, and post-activation periods of the deterrent devices to assess if the devices influenced the rate of visitation using linear models in program R. We analyzed sites with and without the deterrent devices separately to assess the effects of each treatment throughout the days independently.

For the second index, we estimated rates of the number of wild pigs, non-target mammals, and non-target birds, respectively, observed per hour that visited bait sites. We followed methodology established by^22^, and used negative binomial generalized mixed models with package glmmTMB^37^ to compare rates of visitation between periods of pre- and post-SN-toxic bait deployment to assess changes relative to toxic baiting. We considered the change in rates of visitation to be attributed to lethality from SN-toxic bait for the populations of animals visiting the bait sites. We expect this methodology met the assumption that detection of animals remained consistent^47^ at bait sites because pre- and post-toxic periods were only separated by a single 24-h period when the toxic bait was deployed, and we refreshed the bait daily. We also compared the indices between treatments (with vs without deterrents) and the interaction of period × treatment. The models examined for each group of species were: rate of hourly visitation ~ period + treatment + period × treatment. We also used Site ID as random effects to account for repeated measures taken at each bait site.

For the transect analysis, we calculated descriptive summaries of sexes, ages, and distances from carcass to nearest bait station for wild pigs that succumbed to the SN-toxic bait. We also summarized any non-target deaths and distances from the nearest bait site. All research methods for all phases of this study were approved under the USDA National Wildlife Research Center, Institutional Animal Care and Use Committee (protocol QA-3068), and performed and reported in accordance with ARRIVE guidelines and US EPA regulations.

## Results

### Field study on deterrent effectiveness for birds

**Figure 4:**
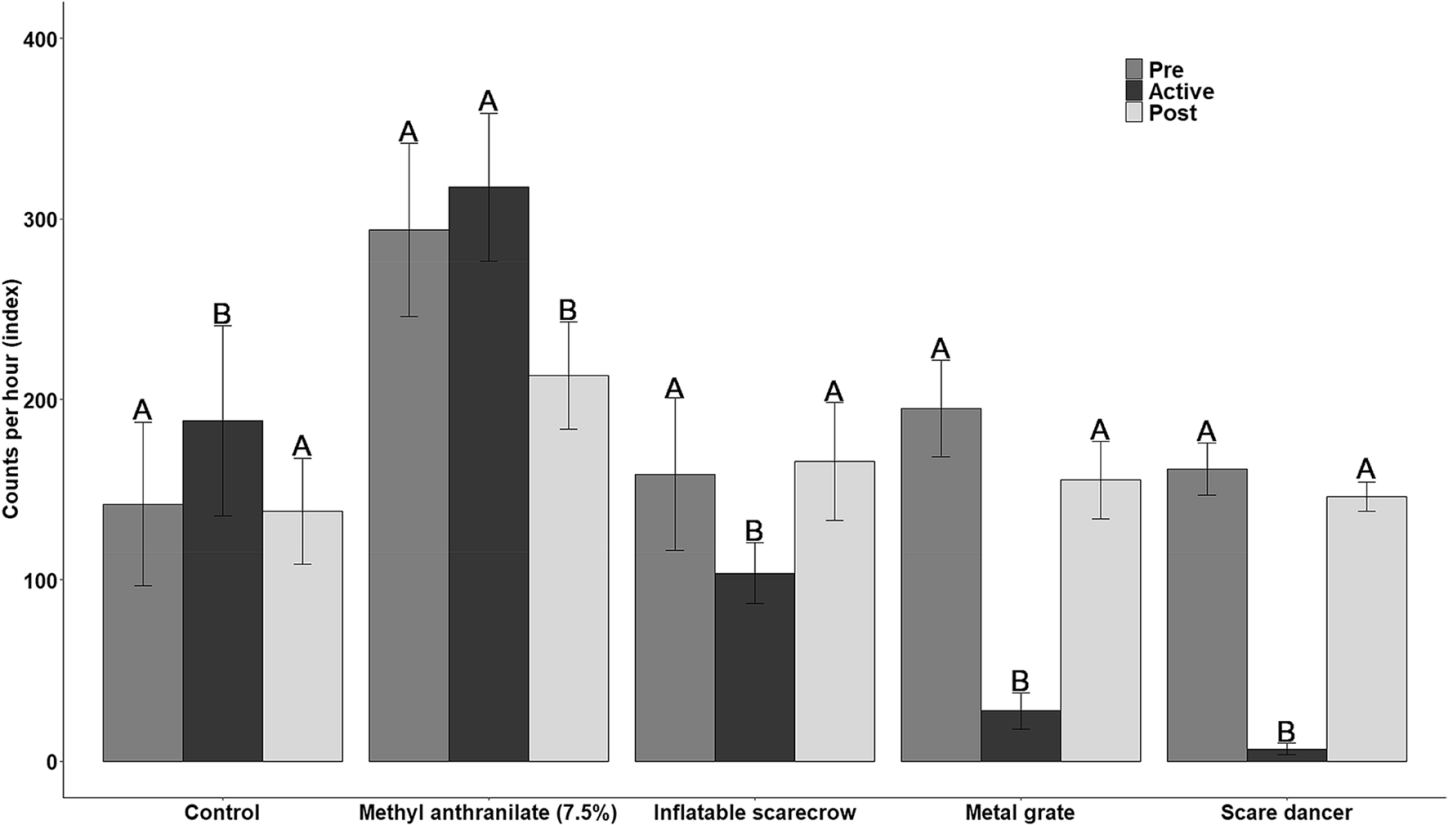
Index of birds observed visiting bait sites pre-, during, and post-activating of potential deterrents in north-central Colorado, USA during April–May 2020. Letters above the bars indicate statistical differences within each treatment group. No toxic bait was applied during this evaluation.

Overall, we identified 46 species visiting bait sites throughout the duration of the study in north-central CO, 32 of which were grain-eating birds which we included in our analyses (Supplemental Table 1). The rate of bait site visitation by these birds averaged 187.5 (SE = 20.1) per hour during pre-test mornings among all bait sites. We found that the scare dancer reduced visitation by birds an average of 95.7% (β = − 3.16; 95% CI − 3.76 to − 2.56) during the test morning (Fig. 4). The metal grate also substantially reduced visitation by an average of 85.7% (β = − 1.96; 95% CI − 2.50 to − 1.43). The inflatable scarecrow reduced visitation to a lesser extent (i.e., an average of 34.5%) but still produced a significant reduction (β = − 0.38; 95% CI − 0.70 to − 0.06). The methyl anthranilate treatment did not reduce visitation (β = 0.08; 95% CI − 0.10 to 0.27). Visitation to the control sites increased slightly (i.e., an average of 19%) during the test morning (β = 0.37; 95% CI 0.08–0.66).

### Effects of deterrents on captive wild pigs

Captive wild pigs in the methyl anthranilate group consumed an average of 23.1% (SE = 5.3) of the corn offered, whereas the control group consumed 24.4% (SE = 3.9). We found no significant interactions of treatment × night, indicating no evidence that methyl anthranilate reduced consumption of corn by wild pigs over time. From the reduced model, we found no difference in consumption of corn between the methyl anthranilate and control groups (β = − 0.14; 95% CI − 0.92 to 0.63). Similarly, we found no difference in consumption on the second (β = − 0.36; 95% CI − 1.32 to 0.59) or third (β = − 0.53; 95% CI − 1.48 to 0.42) nights of the treatments, relative to the first night.

### Field evaluation of deterrent with toxic bait

Overall, we identified 9 species of non-target birds and 3 species of non-target mammals that used bait sites for wild pigs in north-central TX (Supplemental Table 2). There was no difference in the rate of non-target animals/hour visiting bait sites between sites with and without deterrent devices (β = 0.59; 95% CI − 0.89 to 2.06) during pre-toxic baiting, indicating an unbiased comparison between treatments. Similarly, visitation rates of wild pigs/hour during pre-toxic baiting was similar between sites with and without deterrent devices (β = 0.27; 95% CI − 1.32 to 1.86).

**Figure 5:**
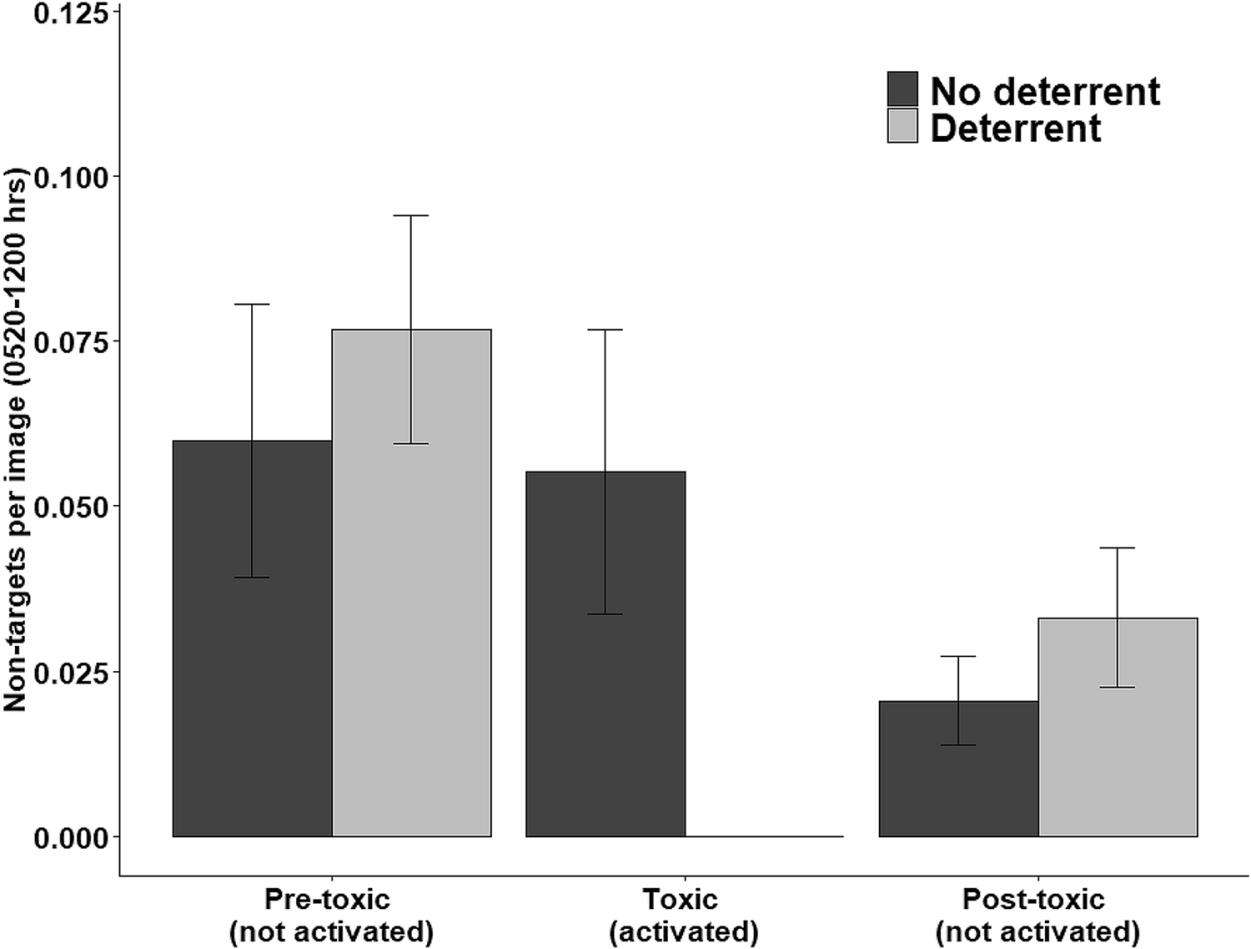
Index of non-target animals (primarily birds) using bait sites pre-, during, and post-activation of a deterrent device during the hours of 0520–1200 at sodium nitrite toxic bait sites in north-central Texas, USA during July 2020.

The deterrent devices were 100% effective during 0520–1200 h (duration of operation) because no non-target animals were observed on camera near bait sites during those hours (Fig. 5). As such, we found a significant decline in the rate of non-targets observed at bait sites on the day the deterrent devices were activated (i.e., the morning after SN-toxic baiting; β = − 0.26; 95% CI − 0.45 to − 0.06), compared to the days pre- and post-activation. Contrarily, we observed no changes in the rate of non-target visitations to the bait sites without deterrent devices (β = − 0.19; 95% CI − 0.41 to 0.03).

**Figure 6:**
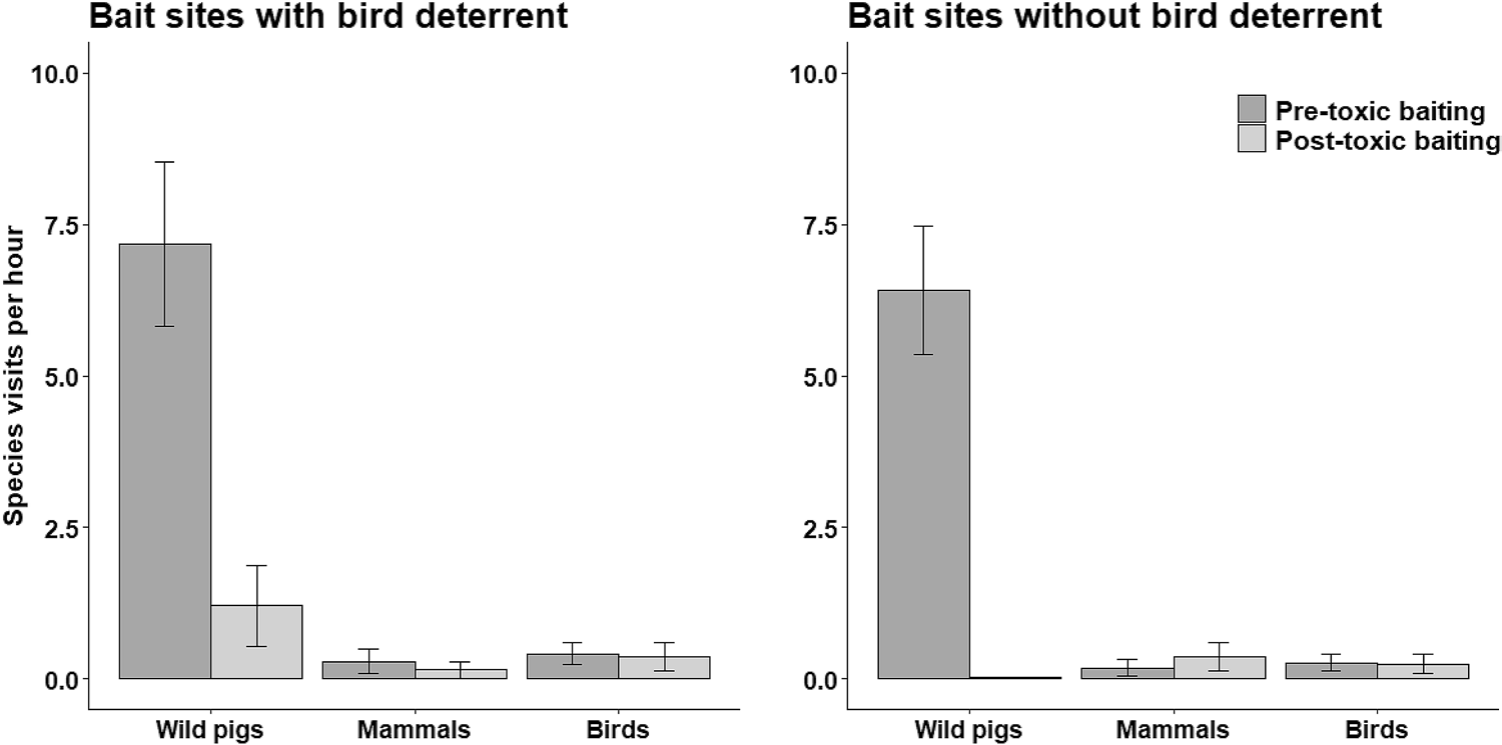
Index of species visitation to SN-toxic bait sites for wild pigs one day pre- and post-toxic baiting in north-central Texas, USA during July 2020.

For the measures of lethality at bait sites, the interaction of period × treatment was not significant (β = − 0.05; 95% CI − 0.79 to 0.70) for non-target birds, indicating no evidence of population declines at either of the bait site treatments (Fig. 6). The main effect for period also showed no evidence of changes in non-target bird visitation pre- and post-toxic baiting (β = − 0.11; 95% CI − 0.67 to 0.45), indicating SN-toxic bait did not cause declines in counts of birds that visited bait sites. For non-target mammals, the model including the interaction of period × treatment would not converge because of low counts of visits by non-target mammals. The simplified model without the interaction term indicated no evidence of changes in the rate of non-target mammals that visited bait sites (β = − 0.16; 95% CI − 1.37 to 1.04) pre- and post-toxic baiting. For wild pigs, the interaction of period × treatment indicated that the rate of wild pigs visiting bait sites was greater at sites with deterrents post-toxic baiting than at sites without deterrents (β = 4.03; 95% CI 1.93–6.13). However, that rate was significantly reduced from pre- to post-toxic baiting at all sites (β = − 5.82; 95% CI − 7.59 to − 4.04). The decline in rates of visitation averaged 91.0% at all bait sites following one night of SN-toxic baiting, including an average of 83.2% decline at sites with deterrent devices, and a 99.7% decline at sites without deterrent devices.

Overall, we located the carcasses of 139 wild pigs (68 females, 64 males, and 7 unknown sex because of scavenging or decomposition) during transect searches. The carcasses of wild pigs were located an average of 226.2 m (SE = 15.6) from the nearest bait site. We found all age classes of wild pigs, including 22 at 8–20 weeks, 47 at 20–30 weeks, 3 at 30–51 weeks, 8 at 12–18 months, 15 at 18–26 months, 11 at 26–36 months, 15 at 36–48 months, 13 at ≥ 48 months, and 5 unknown ages. We also found the carcasses of two non-target animals, including a deer mouse (*Peromyscus maniculatus*) and a plains pocket mouse (*Perognathus flavescens*). Both non-target animals were found at bait sites without the deterrent devices and averaged 31.5 m (SE = 6.0) away from the nearest bait site. We found no carcasses of non-target birds.

## Discussion

In our first objective we identified scare dancer deterrent devices as the most effective deterrent for birds at bait sites. In our final objective we demonstrated success for reducing hazards to birds by using those deterrent devices at SN-toxic bait sites for wild pigs. We expected the deterrent device was highly effective because it was novel, operated continuously in an erratic pattern, and made a snapping sound as it moved. Using the deterrent devices reduced our observations of non-target animal visitation to bait sites by 100% the morning following deployment of the SN-toxic bait, which subsequently reduced hazards to non-target animals. We surmised the deterrent devices lent themself well to the risks from a SN-toxic bait because deterrence was only needed during the early morning hours after wild pigs visited the bait site and operators arrived to clean up any bait spilled by the wild pigs^22,24^. The non-target animals did not have time to acclimate to the devices.

Our results also revealed that seasonality was important for reducing hazards to non-target animals, especially concerning migratory birds. A similar field trail in March 2020 demonstrated that the presence of migrating birds at the same study site in north-central TX resulted in the deaths of 35 birds (mostly dark‐eyed juncos [*Junco hyemalis*]) from consuming spilled toxic bait at five bait sites without a deterrent device present^24^. Whereas during this study (July 2020), we observed no deaths of birds even at the five bait sites without the deterrent devices. Dark-eyed juncos were commonly observed at toxic bait sites during March 2020 but were not observed in July 2020 (Supplemental Table 2). This trend was also evident with white-crowned sparrows (*Zonotrichia leucophrys*) during a similar field test in March 2018 in north-central TX^23^, but were not observed at nearby bait sites during this mid-summer study. Lastly, we found that wild pigs were easily located and baited near water sources during mid-summer, which is another reason for season-specific baiting.

A few downsides exist to using deterrent devices at SN-toxic baiting sites. Primarily, there is a risk that the device could deter wild pigs from consuming the SN-toxic bait. We purposefully programmed the devices to activate as late as possible without increasing risks to non-target birds feeding in the early mornings. This intentional programming coupled with wild pigs visiting bait sites at dusk or nighttime^22^ seemed to mostly alleviate this potential downside. Only at one of five bait sites did we observe wild pigs being scared away when the deterrent devices were activated at 0520 h. These wild pigs had already consumed a lethal dose of the SN-toxic bait though and we found their carcasses. To our knowledge, all other wild pigs visited bait sites earlier in the evenings and were likely dead by the time the deterrent devices were activated (i.e., time to death following consumption of a letahl dose = 2–3 h^10^). However, we did find slightly lower lethality for wild pigs at sites with the deterrent devices, indicating that possibly the devices deterred wild pigs from consuming SN-toxic bait. This could also be an artifact of testing only five bait sites with and without the deterrent devices. Regardless of the reason, deploying the SN-toxic bait for a second night could increase the lethality, but we were not authorized to do this by the US EPA for this study. Other potential downsides are the additional costs, including the price of the device (~ $99.95 USD in May 2020), the price of a 12v deep cycle battery (~ $108.50 USD; Duracell SLI31MDC, Batteries + Bulbs, Fort Collins, CO, USA), and the additional labor time for setting up and taking down the device (~ 30–60 min total).

The other deterrent devices we tested were not suitable to reducing hazards for non-target animals at SN-toxic bait sites. Although the metal grate successfully protected bird seed underneath the grate from birds, we found that some particles of a peanut paste-based, placebo SN-toxic bait would stick to the top of the grate and still be available for non-target animals to consume. Additionally, wild pigs would have to walk on top of the metal grate which we observed some aversion too, thus may require more acclimation time. The inflatable scarecrow did not appear to operate continuously enough (i.e., spans of 18 min without activity) to successfully keep non-target birds away. This device was also found to be less effective than other deterrents in previous research^48^. Finally, the maximum concentration of methyl anthranilate (7.5%) specified on the product label sprayed on the ground did not appear to deter birds, which was surprising considering we applied this concentration for three consecutive days. However, methyl anthranilate has demonstrated mixed effectiveness in previous studies^29,30,49^ and simply may not be aversive enough to deter birds from bait sites in which birds are highly acclimated to visiting. Methyl anthranilate did not appear to deter wild pigs either, which may suggest some potential to protect non-target birds if a different strategy was found to be more effective than the label-specified spot-treatment we employed.

This study provides promise for the continued development of a SN-toxic bait for wild pigs, though it also has some limitations. Primarily, we did not evaluate the deterrent device during the late-winter/early-spring season when migrating birds were present in north-central TX study area. This was intentional as we also wanted to evaluate whether seasonality reduced non-target hazards. Testing the deterrent device at SN-toxic bait sites during the late-winter/early-spring would be important if toxic baiting is planned for those seasons. Additionally, we recognize that monitoring of bait sites with remote cameras from 5 m away is not sufficient for detecting small rodents that might use bait sites. The deterrent devices and careful removal of any spilled bait should minimize hazards to rodents, but more investigation is needed possibly using remote cameras at closer distances or using video cameras. Finally, we were only authorized (by US EPA) to test 10 toxic bait sites during this study which is a small sample size and our implications should be viewed with caution. However, based on our results, and the results of our other recent studies on other aspects of SN-toxic bait development for wild pigs, US EPA has granted an Experimental Use Permit to conduct more comprehensive evaluations with larger samples sizes during summer 2021 in TX and AL.

### Management implications

Our results revealed two strategies for reducing hazards to non-target birds at SN-toxic bait sites for wild pigs. First, we recommend deploying a deterrent device (programmable, battery operated, continuous and erratic movement, and beating sounds) at bait sites, and activating it very early in the morning, before birds start feeding. Second, we recommend deploying SN-toxic bait during seasons when migrating birds are not present until further research is conducted during more risky seasons. Many of the bird species that revealed hazards in previous late-winter/early-spring SN-trials (e.g., white-crowned sparrows and dark-eyed juncos) were not present during mid-summer in north-central TX. Using either or both strategies substantially reduced hazards to non-target species. We currently recommend employing both strategies because the additional expense and labor of including the deterrent device is not too burdensome and provides an additional level of protection to non-target birds, and wild pigs can be baited successfully during summertime at limited water sources.

## Supplementary Information

Supplementary Information.

## References

[1] Bevins SN, Pedersen K, Lutman MW, Gidlewski T, Deliberto TJ (2014). Consequences associated with the recent range expansion of nonnative feral swine. Bioscience.

[2] Mayer, J. & Brisbin, I. L. (Savannah River National Laboratory, 2009).

[3] Barrios-Garcia MN, Ballari SA (2012). Impact of wild boar (*Sus scrofa*) in its introduced and native range: A review. Biol. Invasions.

[4] Lowe, S., Browne, M., Boudjelas, S. & De Poorter, M. *100 of the world's worst invasive alien species: a selection from the global invasive species database* (The Invasive Species Specialist Group (ISSG) a specialist group of the Species Survival Commission (SSC) of the World Conservation Union (IUCN), 2000) http://www.issg.org/booklet.pdf.

[5] Pimentel, D. In *Managing Vertebrate Invasive Species: Proceedings of an International Symposium* (eds Witmer, G. W. *et al.*) 2–8 (U.S. Department of Agriculture, Animal and Plant Health Inspection Service, Wildlife Services, National Wildlife Research Center, 2007).

[6] Anderson A, Slootmaker C, Harper E, Holderieath J, Shwiff SA (2016). Economic estimates of feral swine damage and control in 11 US states. Crop Protect..

[7] Snow NP, Jarzyna MA, VerCauteren KC (2017). Interpreting and predicting the spread of invasive wild pigs. J. Appl. Ecol..

[8] Lewis JS (2019). Historical, current, and potential population size estimates of invasive wild pigs (*Sus scrofa*) in the United States. Biol. Invasions.

[9] Lewis JS (2017). Biotic and abiotic factors predicting the global distribution and population density of an invasive large mammal. Sci. Rep..

[10] Snow NP (2017). Development of toxic bait to control invasive wild pigs and reduce damage. Wildl. Soc. Bull..

[11] Poché RM (2019). Development of a low-dose warfarin bait for controlling feral hogs. Crop Protect..

[12] Lapidge, S. *et al. *Development of a feral swine toxic bait (Hog-Gone) and bait hopper (Hog-Hopper™) in Australia and the USA. In *Proceedings of the 14th Wildlife Damage Management Conference*, Vol. 14 (2012).

[13] Shapiro L (2016). Efficacy of encapsulated sodium nitrite as a new tool for feral pig management. J. Pest Sci..

[14] Jori F (2020). Application of the World Café method to discuss the efficiency of African swine fever control strategies in European wild boar (*Sus scrofa*) populations. Prev. Vet. Med..

[15] Beasley JC, Ditchkoff SS, Mayer JJ, Smith MD, Vercauteren KC (2018). Research priorities for managing invasive wild pigs in North America. J. Wildl. Manag..

[16] Snow NP (2016). Bait preference of free-ranging feral swine for delivery of a novel toxicant. PLoS ONE.

[17] Snow NP (2018). Potential secondary poisoning risks to non-targets from a sodium nitrite toxic bait for invasive wild pigs. Pest Manag. Sci..

[18] Snow NP (2019). Low secondary risks for captive coyotes from a sodium nitrite toxic bait for invasive wild pigs. Wildl. Soc. Bull..

[19] Shapiro L, Blackie H, Arthur D, Ross J, Eason C (2018). Secondary poisoning risk for encapsulated sodium nitrite, a new tool for possum control. N. Z. J. Ecol..

[20] Lavelle MJ (2018). Development and evaluation of a bait station for selectively dispensing bait to invasive wild pigs. Wildl. Soc. Bull..

[21] Snow NP (2017). Strength testing of raccoons and invasive wild pigs for a species-specific bait station. Wildl. Soc. Bull..

[22] Snow NP (2021). Daily and landscape influences of species visitation to toxic bait sites for wild pigs. Wildl. Soc. Bull..

[23] United States Department of Agriculture. *APHIS Wildlife Services conducts first field trial of feral swine toxic bait; plans modifications to mitigate hazards to non-target species* (2018).

[24] Snow NP, Wishart JD, Foster JA, Staples LD, VerCauteren KC (2021). Efficacy and risks from a modified sodium nitrite toxic bait for wild pigs. Pest Manag. Sci..

[25] Gilsdorf JM, Hygnstrom SE, VerCauteren KC (2002). Use of frightening devices in wildlife damage management. Integr. Pest Manag. Rev..

[26] Avery, M. L. & Werner, S. J. In *Ecology and Management of Blackbirds (Icteridae) in North America* Vol. 9 (eds Linz,G. M. *et al.*) Ch. 9, 159–176 (CRC Press, 2017).

[27] Tillman, E. A., Van Doom, A. & Avery, M. L. In *The Ninth Wildlife Damage Management Conference Proceedings* (eds Brittingham, M. C. *et al.*) 47–59 (2000).

[28] Fuller-Perrine LD, Tobin ME (1993). A method for applying and removing bird-exclusion netting in commercial vineyards. Wildl. Soc. Bull..

[29] Avery ML (1995). Methyl anthranilate as a rice seed treatment to deter birds. J. Wildl. Manag..

[30] Werner, S. J. & Avery, M. L. In *Ecology and management of blackbirds (Icteridae) in North America* (eds Linz, G. M. *et al.*) Ch. 8, 135–158 (CRC Press, 2017).

[31] Curtis PD, Merwin IA, Pritts MP, Peterson DV (1994). Chemical repellents and plastic netting for reducing bird damage to sweet cherries, blueberries, and grapes. HortScience.

[32] Cooke, W. W. (ed Ellis, A.) 143 (Bulletin No. 37, Technical Series No. 2, State Agricultural College, Agricultural Experiment Station, 1897).

[33] Chapman S (2006). Ecoregions of Colorado.

[34] Bailey, R. G. *Description of the ecoregions of the United States* (US Department of Agriculture, Forest Service, 1980).

[35] Bailey RG (1998). Ecoregions, the Ecosystem Geography of the Oceans and Continents.

[36] Kare, M. R. Bird Repellent. *US Patent 2967128* (1961).

[37] Magnusson, A. *et al. Generalized Linear Mixed Models using Template Model Builder* (2020).

[38] R Core Team. *R Foundation for Statistical Computing* (2020).

[39] Engqvist L (2005). The mistreatment of covariate interaction terms in linear model analyses of behavioural and evolutionary ecology studies. Anim. Behav..

[40] Lavelle MJ (2018). Evaluation of movement behaviors to inform toxic baiting strategies for invasive wild pigs (*Sus scrofa*). Pest Manag. Sci..

[41] Snow NP, VerCauteren KC (2019). Movement responses inform effectiveness and consequences of baiting wild pigs for population control. Crop Protect..

[42] Snow NP (2019). Exposure of a population of invasive wild pigs to simulated toxic bait containing biomarker: Implications for population reduction. Pest Manag. Sci..

[43] McRae JE (2019). Factors affecting bait site visitation: Area of influence of baits. Wildl Soc. Bull..

[44] Halseth, J. M., Lavelle, M. J., Snow, N. P. & VerCauteren, K. C. Technical note: aging feral swine in the field (USDA/APHIS/WS/National Wildlife Research Center, 2018).

[45] Patton TG, Blamer SL, Horak KE (2016). Detecting methemoglobinemia in animals with a drop of blood. PLoS ONE.

[46] Ivan JS, Newkirk ES (2016). CPW Photo Warehouse: A custom database to facilitate archiving, identifying, summarizing and managing photo data collected from camera traps. Methods Ecol. Evol..

[47] Pollock KH (2002). Large scale wildlife monitoring studies: Statistical methods for design and analysis. Environmetrics.

[48] Andelt WF, Woolley TP, Hopper SN (1997). Effectiveness of barriers, pyrotechnics. Wildl. Soc. Bull..

[49] Avery, M. L. & Cummings, J. L. In *Management of North American Blackbirds (Proceedings of a special symposium of the Wildlife Society 9th annual Conference)* (ed Linz, G. M.) 41–48 (2003).

