# Supplementary material for: Deterring non-target birds from toxic bait sites for wild pigs
Source: Sci Rep. 2021 Oct 7;11:19967. doi: 10.1038/s41598-021-99547-8 (PMC8497612; doi:10.1038/s41598-021-99547-8)
Supplement: Supplementary file 1 — Supplementary Information. [file 41598_2021_99547_MOESM1_ESM.docx]

**Supplemental Table 1.** Average count of species observed at bait sites using 2 kg of bird seed (Wild Birds Unlimited Deluxe Blend, Fort Collins, CO, USA) refreshed daily in north-central Colorado, USA during April 2020.

| **Species** | **Average count per hr** | **SE** |
| --- | --- | --- |
| **GRAIN-EATING BIRDS** |  |  |
| Red-winged black bird (*Agelaius phoeniceus*) | 134.14 | 7.78 |
| White-crowned sparrow (*Zonotrichia leucophrys*) | 6.58 | 1.39 |
| Mourning dove (*Zenaida macroura*) | 2.75 | 0.62 |
| Common grackle (*Quiscalus quiscula*) | 1.60 | 0.29 |
| House finch (*Haemorhous mexicanus*) | 1.34 | 0.38 |
| Blue jay (*Cyanocitta cristata*) | 1.29 | 0.19 |
| Yellow-headed blackbird (*Xanthocephalus xanthocephalus*) | 0.92 | 0.24 |
| Black-billed magpie (*Pica hudsonia*) | 0.72 | 0.12 |
| Brown-headed cowbird (*Molotrhus ater*) | 0.52 | 0.07 |
| House sparrow (*Passer domesticus*) | 0.30 | 0.14 |
| Wild turkey (*Meleagris gallopavo*) | 0.20 | 0.10 |
| Spotted towhee (*P. maculatus*) | 0.14 | 0.05 |
| Eurasian collared dove (*Streptopelia decaocto)* | 0.11 | 0.02 |
| Green-tailed towhee (*Pipilo chlorurus*) | 0.05 | 0.02 |
| Brown thrasher (*Toxostoma rufum*) | 0.05 | 0.03 |
| Black-capped chickadee (*Poecile atricapillus*) | 0.04 | 0.01 |
| American goldfinch (*Spinus tristis*) | 0.04 | 0.02 |
| Canada goose (*Branta canadensis*) | 0.04 | 0.03 |
| Chipping sparrow (*Spizella passerina*) | 0.03 | 0.02 |
| Lark sparrow (*Chondestes grammacus*) | 0.03 | 0.03 |
| Lazuli bunting (*Passerina amoena*) | 0.02 | 0.01 |
| White-breasted nuthatch (*Sitta carolinensis*) | 0.02 | 0.01 |
| Song sparrow (*Melospiza melodia*) | 0.02 | 0.02 |
| Dark-eyed junco (*Junco hyemalis*) | 0.02 | 0.01 |
| Lincoln's sparrow (*M. lincolnii*) | 0.01 | 0.01 |
| Common pheasant (*Phasianus colchicus*) | 0.004 | 0.004 |
| Common starling (*Sturnus vulgaris*) | 0.003 | 0.002 |
| American robin (*Turdus migratorius*) | 0.003 | 0.002 |
| Woodhouse's scrub jay (*Aphelocoma woodhouseii*) | 0.002 | 0.002 |
| Unknown sparrows *Passeridae* | 0.002 | 0.001 |
| Western meadowlark (*Sturnella neglecta*) | 0.002 | 0.001 |
| Red-breasted nuthatch (*Sitta canadensis*) | 0.002 | 0.001 |
| Rose-breasted grosbeak (*Pheucticus ludovicianus*) | 0.001 | 0.001 |
| **OTHER BIRDS** |  |  |
| Downy woodpecker (*Picoides pubescens*) | 0.04 | 0.01 |
| Bullock's oriole (*Icterus bullockii*) | 0.002 | 0.001 |
| Hairy woodpecker (*Leuconotopicus villosus*) | 0.002 | 0.002 |
| House wren (*Troglodytes aedon*) | 0.001 | 0.001 |
| Western kingbird (*Tyrannus verticalis*) | 0.001 | 0.001 |
| **MAMMALS** |  |  |
| Fox squirrel (*Sciurus niger*) | 2.79 | 0.47 |
| Deer species (*Odocoileus* spp.) | 0.59 | 0.08 |
| Raccoon (P*rocyon lotor*) | 0.15 | 0.05 |
| American badger (*Taxidea taxus*) | 0.02 | 0.01 |
| Striped skunk (*Mephitis mephitis*) | 0.003 | 0.003 |
| Coyote (*Canis latrans*) | 0.003 | 0.003 |
| Eastern cottontail (*Sylvilagus floridanus*) | 0.003 | 0.002 |
| Feral cat (*Felis catus*) | 0.002 | 0.001 |

**Supplemental Table 2.** Average count of species observed per hour at 10 bait sites for deploying a sodium nitrite toxic bait to wild pigs during a 24 hour-period pre- and post-toxic baiting in north-central Texas, USA, July 2020.

|  | Pre-toxic baiting | |  | Post-toxic bating | |
| --- | --- | --- | --- | --- | --- |
| Species | Average count per hour | SE |  | Average count per hour | SE |
| **NON-TARGET BIRDS** |  |  |  |  |  |
| Lark sparrow (*Chondestes grammacus*) | 0.187 | 0.10 |  | 0.145 | 0.08 |
| Northern cardinal (*Cardinalis cardinalis*) | 0.068 | 0.05 |  | 0.066 | 0.06 |
| Northern bobwhite quail (*Colinus virginianus*) | 0.044 | 0.03 |  | 0.027 | 0.02 |
| Mourning dove (*Zenaida macroura*) | 0.022 | 0.01 |  | 0.022 | 0.01 |
| American crow (*Corvus brachyrhynchos*) | 0.015 | 0.02 |  | 0.020 | 0.02 |
| Northern mockingbird (*Mimus polyglottos*) | 0.004 | 0.00 |  | 0.005 | 0.00 |
| Tufted titmouse (*Baeolophus bicolor*) | 0.000 | 0.00 |  | 0.005 | 0.00 |
| Golden-fronted woodpecker (*Melanerpes aurifrons*) | 0.000 | 0.00 |  | 0.014 | 0.01 |
| House wren (*Troglodytes aedon*) | 0.000 | 0.00 |  | 0.005 | 0.00 |
| **NON-TARGET MAMMALS** |  |  |  |  |  |
| Raccoon (*Procyon lotor*) | 0.217 | 0.11 |  | 0.223 | 0.11 |
| Eastern cottontail (*Sylvilagus floridanus*) | 0.015 | 0.02 |  | 0.031 | 0.03 |
| Mule deer (*Odocoileus hemionus*) | 0.008 | 0.01 |  | 0.004 | 0.00 |
| **WILD PIGS** |  |  |  |  |  |
| Wild pig (*Sus scrofa*) | 6.786 | 0.82 |  | 0.613 | 0.37 |
